# Tele-Ultrasound in Resource-Limited Settings: A Systematic Review

**DOI:** 10.3389/fpubh.2019.00244

**Published:** 2019-09-04

**Authors:** Noel Britton, Michael A. Miller, Sami Safadi, Ariel Siegel, Andrea R. Levine, Michael T. McCurdy

**Affiliations:** ^1^University of Pittsburgh Graduate School of Public Health, Pittsburgh, PA, United States; ^2^University of Pittsburgh Medical Center, Pittsburgh, PA, United States; ^3^University of Maryland School of Medicine, Baltimore, MD, United States

**Keywords:** telemedicine, eHealth, ultrasound, resource-limited, tele-ultrasound, LMIC, tele-radiology, global health

## Abstract

**Background:** Telemedicine, or healthcare delivery from a distance, has evolved over the past 50 years and helped alter health care delivery to patients around the globe. Its integration into numerous domains has permitted high quality care that transcends obstacles of geographic distance, lack of access to health care providers, and cost. Ultrasound is an effective diagnostic tool and its application within telemedicine (“tele-ultrasound”) has advanced substantially in recent years, particularly in high-income settings. However, the utility of tele-ultrasound in resource-limited settings is less firmly established.

**Objective:** To determine whether remote tele-ultrasound is a feasible, accurate, and care-altering imaging tool in resource-limited settings.

**Data Sources:** PubMed, MEDLINE, and Embase.

**Study Eligibility Criteria:** Twelve original articles met the following eligibility criteria: full manuscript available, written in English, including a direct patient-care intervention, performed in a resource-limited setting, images sent to a remote expert reader for interpretation and feedback, contained objective data on the impact of tele-ultrasound.

**Study Appraisal and Synthesis Methods:** Abstracts were independently screened by two authors against inclusion criteria for full-text review. Any discrepancies were settled by a senior author. Data was extracted from each study using a modified Cochrane Consumers and Communication Review Group's data extraction template. Study bias was evaluated using the ROBINS-I tool.

**Results:** The study results reflect the diverse applications of tele-ultrasound in low-resource settings. Africa was the most common study location. The specialties of cardiology and obstetrics comprised most studies. Two studies primarily relied on smartphones for image recording and transmission. Real-time, rather than asynchronous, tele-ultrasound image interpretation occurred in five of the 12 studies. The most common outcome measures were image quality, telemedicine system requirements, diagnostic accuracy, and changes in clinical management.

**Limitations:** The studies included were of poor quality with a dearth of randomized control trials and with significant between study heterogeneity which resulted in incomplete data and made cross study comparison difficult.

**Conclusions and Implications of Key Findings:** Low-quality evidence suggests that ultrasound images acquired in resource-limited settings and transmitted using a telemedical platform to an expert interpreter are of satisfactory quality and value for clinical diagnosis and management.

## Introduction

### Background and Rationale

Global health encompasses both research and action aimed at promoting health for all persons, independent of national boundaries (1). Common barriers to global health initiatives include lack of healthcare access and lack of resources (2). Telemedicine (also called mHealth, telehealth, e-health), or literally “healing at a distance,” is a tool well-suited to reduce these barriers (3). The term telemedicine specifically refers to care provided by a physician whereas telehealth is a more global term that encapsulates care provided by all healthcare professionals (e.g., pharmacists, nurses) (4). While its manifestation and implementation can vary across different medical specialties, telemedicine universally attempts to utilize technology to provide clinical support to patients across geographical barriers in an effort to improve patient health outcomes (2). Telemedicine, therefore, functionally expands patient access to care by mitigating geographic barrier to healthcare (5).

The history of telemedicine dates back more than a century. An article published in the *Lancet* in 1879 describes the use of the telephone to reduce patient office visits (6). In the 1900's there were there are reports about physicians using the radio to make a medical diagnosis. In 1906, a paper was published by Willem Einthoven, the inventor of the electrocardiogram, about the use of tele-cardiogram. Einthoven used the telephone cable to transmit a signal from the hospital to his laboratory, 1.5 km away. He subsequently utilized telecardiogram to remotely analyze clinical EKGs from patients in the hospital. By the 1920's, telemedicine provided medical consultation from medical centers in Italy, Norway and France to patients aboard ships and on remote islands (6, 7). By the 1950's, the transmission of radiographic images began in the United States and occurred shortly thereafter in Canada (8). The United States National Aeronautics and Space Administration's (NASA) adopted telemedicine in the 1960's in an effort to ensure safety in space flight. What began as remote monitoring of biometric data in the 1960s gradually escalated to ensure that astronauts could receive an accurate diagnosis by onboard crewmates in the event of a medical emergency. NASA ultimately developed a terrestrial parallel program called Space Technology Applied to Rural Papago Health Care (STARPAHC) (9). In collaboration with the Tohono O'odham tribe of Southern Arizona and the Indian Health Service, NASA used rudimentary telemedicine technology to successfully connect patients in resource-limited areas with physicians at hospitals elsewhere in the state via mobile support units (10). Since then, the field of telemedicine has evolved rapidly, propelled by major technological advances including email, mobile phones, the internet, ultrasound technologies, videoconferencing, and smartphones.

As telemedicine evolved, the field of ultrasonography matured in parallel. By the 1990s, ultrasound technology had developed into a bedside tool that physicians, particularly emergency physicians, were routinely utilizing (11). Ultrasound is a safe (non-ionizing) and portable tool capable of being used in a diagnostic or interventional capacity. Ultrasound has both 2D and 3D capabilities, can be analyzed in real-time, and is a comparatively low-cost imaging modality (12). Moreover, a growing body of evidence demonstrates that bedside ultrasound is more accurate than conventional physical exam for cardiovascular diagnoses (13). In low- and middle-income countries (LMICs), ultrasound and plain radiographs are often the only available imaging modalities (14). As ultrasound machines became increasingly portable and as technologies to support data transmission became commercially available, adequate infrastructure could support the emergence of tele-ultrasound. The tele-ultrasound paradigm involves performing bedside ultrasound at one location with images transmitted and interpreted by a provider located in a geographically distant location. This process can be conducted either in a synchronous, or real-time manner, or in an asynchronous manner. Asynchronous tele-ultrasound utilizes a store-and-forward technique in which images are captured, stored, and later transmitted for image interpretation. Tele-ultrasound offered a seamless solution for skeptics of telemedicine who questioned the ability to ascertain a meaningful physical examination from afar.

Studies based in high-income countries suggest that tele-ultrasound is clinically valuable. Tele-ultrasound has been successfully used in diverse settings, including telecardiology consultation for neonatal units in Northern Ireland, airplanes in flight, Antarctic research stations, even at the International Space Station (15–18). Furthermore, studies have clearly demonstrated that images can be reliably transmitted between geographically distinct locations without loss of clinically important image quality via commercially available two-way audiovisual technology (19–21). Instrumental to the evolution and global utilization of tele-ultrasound was the finding that minimally trained sonographers can acquire high quality images using real-time guidance from experts afar, an infrastructure called remote tele-mentored ultrasound (RTMUS) (20, 22). RTMUS utilizes a single centrally-located physician trained in bedside ultrasound who guides a geographically-removed bedside provider in image acquisition and performs image interpretation from afar. Early work in high-income countries demonstrated that remote tele-mentored ultrasound was feasible and accurate in cardiac, trauma, and critical care applications (22–25).

### Objective

Tele-ultrasound is increasingly used to provide global health care. Even in high-income countries, patient care is frequently limited by a lack of access to trained clinicians. This supply-demand mismatch is further exaggerated in resource-limited settings where a dearth of subspecialty and procedurally-trained physicians often exists and the resources available to those physicians may be limited by economic constraints. The use of tele-ultrasound in resource-limited countries is, therefore, a rapidly burgeoning field. Due to the topic's clinical significance, a need exists to aggregate the various studies on the topic of tele-ultrasound in resource-limited settings. The goal of this paper is to systematically review the literature to determine whether remote tele-ultrasound is a feasible and accurate imaging modality that alters the care provided to patients in resource-limited settings compared to the standard of care. To our knowledge, no prior systematic review has been conducted on this topic.

## Methods

### Design and Study Selection

We performed a review of all published reports of tele-ultrasound in resource-limited settings. This review follows the PRISMA guidance for systematic reviews (26). We included full manuscripts written in the English language and we excluded non-human studies, studies using exclusively 1D ultrasound, review articles, abstracts, case reports, and editorials. Inclusion criteria required: (1) a direct patient-care intervention; (2) performance in a resource-limited setting; (3) patient ultrasound images sent to a remote, expert reader for interpretation and feedback; and (4) objective data on the clinical impact of tele-ultrasound. In this study, we defined resource-limited settings as low-resource areas in LMICs. We excluded studies conducted in remote areas of resource-abundant countries. Studies that involved images collected by robotic arm or under the aide of virtual reality technologies were excluded. Only studies published before January 1, 2019 were included.

### Search Strategy

The literature search was conducted under the direction of the University of Maryland Health Sciences and Human Services Library Systematic Review Consultation Service (Baltimore, MD, USA). Databases searched include PubMed, MEDLINE, and Embase. Search terms included “ultrasound” AND “telemedicine” AND “resource-limited” present in the title or abstract, as well as common synonyms, including sonography, eHealth, developing world, and more (detailed electronic search strategy including relevant MESH terms for PubMed and MEDLINE and Emtree/exploded terms for Embase in Supplementary Materials 1.1 and 1.2, respectively). Relevant MESH and Emtree/exploded terms were also included. The database searches were completed on February 1, 2019 with all manuscripts published prior to January 1, 2019 evaluated for eligibility for inclusion in this review. Next, we searched the references of included papers to identify additional studies meeting inclusion criteria.

Two authors independently screened abstracts and selected candidate articles for full text review. If either author wanted to include a study for full text review, the full text was reviewed in its entirety. Full text review of remaining studies based on inclusion and exclusion criteria identified the final group of studies. A senior author settled any discrepancy in article selection between the two initial authors.

### Data Extraction and Analysis

Data extracted from each study included: study type, study location, publication year, tele-ultrasound method (real-time vs. asynchronous), sample size, patient demographics, organ system assessed, available cost data, ultrasound performer training level, interpreter training level and location, ultrasound type, telemedicine platform, and clinical outcomes. Descriptive statistics were used to report trends in the performer training level, specialty, and outcome measured.

### Bias Assessment

We utilized a tool adapted from the ROBINS-I from the Cochrane collaboration in order to evaluate bias at an individual study level (Table 1) (39). The quality of the studies included in this systematic review was poor which precluded any further quantitative data analysis.

**Table 1:** Assessment of bias in individual papers.

**Study**	**Potential bias**	**Risk of bias**	**Support for judgement**
Adambounou et al. (27)	(1) Bias in selection of participants into the study	(1) No information	(1) Details of participant selection including inclusion and exclusion criteria are not provided for participants undergoing US or for participants performing US
	(2) Bias in measurement of outcomes	(2) Serious	(2) Image quality and diagnoses were assessed by a single expert radiologist; Standard scoring mechanism for image quality was not utilized
Adambounou et al. (28)	(1) Bias in selection of participants into the study	(1) No information	(1) Details of selection for non-physician participants performing US are not provided: “With inexperienced ultrasound operators at CHR Tsévie (e.g., radio operators, nurses, midwives), 10 delayed-time diagnostic tele-ultrasound cases were performed with the virtual navigation program ECHO-CNES.”; Details of selection for participants undergoing US are lacking in precision: “Patients gave full informed consent. These patients were either recruited upon emergency admission to hospital or were already hospitalized at CHR Tsévie.”
	(2) Bias in measurement of outcomes	(2) Moderate	(2) A description of how image quality was standardized and assessed by experts is not provided: “The quality of the images tele-transmitted were appreciated by three expert radiologists (University hospital radiologist), the appreciation retained for the quality of the transmitted images for every bandwidth was that of at least two of the three expert radiologists.”
	(3) Bias in classifications of interventions	(3) Serious	(3) US were performed by both experienced physicians and inexperienced ultrasound operators. Comparing image quality between these two different groups of ultrasound operators compromises the internal validity of the study: “With inexperienced ultrasound operators at CHR Tsévie (e.g., radio operators, nurses, midwives), 10 delayed-time diagnostic tele-ultrasound cases were performed with the virtual navigation program ECHO-CNES.”
Bagayoko et al. (29)	(1) Bias in selection of participants into the study	(1) No information	(1) Details of selection for non-physician/non-midwife participants performing US are not provided: “For the shifting of these tasks in ultrasound imaging and cardiology, a 3 week training of health care professionals was held in Bamako in order to develop basic technical skills.”; Details of selection of study sites are not provided: “Our study was conducted in district hospitals in Bank- ass, Dioila, Kolokani, and Djenne, in rural Mali.”; Details of eligibility criteria for participants undergoing ultrasound are not provided: “Between March 2012 and March 2013, study participants presenting to the one of the four district hospitals with an obstetrical or cardiac problem were invited to participate and were enrolled prospectively to the study.”
	(2) Bias in measurement of outcomes	(2) Serious	(2) Data regarding the impact on care were collected using an unvalidated questionnaire. It is unclear if more than one physician validated these questionnaire results: “The medical evaluation questionnaires were completed by physicians after the consultation with the patient.”
	(3) Bias in classifications of interventions	(3) Critical	(3) Data regarding the use of EKG and US were presented together as one intervention rather than being separated out from each other. As these two interventions are very different, this aggregation could create a critical lack of internal validity.
	(4) Bias due to confounding	(4) Moderate	(4) Clinical sites were chosen to serve as study sites or control sites for outcomes regarding impact on attendance in health centers. No information was provided about the allocation of clinics to either study site or control. No information was provided about the demographic characteristics of the clinics to allow for comparison. The possibility for significant confounding factors to bias results between clinical sites exists.
	(5) Bias due to missing data	(5) Moderate	(5) There were a significant number of missing data regarding impact on care: “The sample consisted of 215 participants for the first indicator, 103 for the second, and 211 for the last.”
Bhavnani et al. (30)	(1) Bias due to participant selection	(1) Moderate	(1) Details of selection for physician participants are not provided: “Five cardiologists and 12 sonographers from 12 academic medical centers across the United States, 15 cardiologists and cardiothoracic surgeons from SSSIHMS, and 30 cardiologists from across India participated in the study.”
	(2) Bias due to confounding	(2) Low	(2) Details of the randomization of clinical sites to mHealth or standard-care are not provided: “Study subjects were evaluated in either 1 of 10 (5 mHealth, or 5 standard care) clinical sites all located at SSSIHMS.”
	(3) Bias due to missing data	(3) Low	(3) There was minimal missing data at 12-month follow up (mHealth: 7%, standard-care: 8%). However, the rates of those lost to follow up were nearly identical in each intervention group.
	(4) Bias due to measurement of outcomes	(4) Low	(4) All testing was performed according to standardized and validated protocols diminishing bias. mHealth devices were subject to daily quality control testing. Results of testing were interpreted by a single physician; however surgical decision making was performed by blinded physicians. Additionally, all results were adjudicated by the primary investigators.: “The primary investigators at SSSIHMS adjudicated all clinical endpoints and determined the necessity for percutaneous or surgical treatment … Subsequently, operating interventional cardiologists and surgeons (different than those performing the initial assessment) were blinded to a study subject's group allocation; however, they reviewed the findings on TTE for diagnostic accuracy at the time of planned percutaneous intervention or surgical procedures.”
Epstein et al. (31)	(1) Bias due to participant selection	(1) No information	(1) Details of participant selection including inclusion and exclusion criteria are not provided for participants undergoing US. The potential for selecting participants with more acute illness is very possible: “Over a 14-day period, 23 of the 75 (30%) acutely ill patients received, by clinical indication, augmented physical examination using pocket size ultrasound machine.”
	(2) Bias in measurement of outcomes	(2) Serious	(2) The initial diagnosis and POCUS were performed by a physician in training and were then confirmed by a single experienced physician: “The studies were performed over a period of 14 days by an internal medicine resident, who was providing volunteer medical care as part of the Israeli Medicine on the Equator project. All studies were conducted for clinical indications … all the studies were reviewed by experienced radiologists and cardiologists, who were all in agreement with the treating physician.”
Martinov et al. (32)	(1) Bias due to participant selection	(1) No information	(1) Details of participant selection including inclusion and exclusion criteria are not provided for participants undergoing US.
	(2) Bias in measurement of outcomes	(2) Moderate	(2) The still images were reviewed by 5 experienced clinicians; however, the video footage was reviewed by a separate single reviewer only: “An image database was created by 50 transmitted images. Five experienced clinicians from Children's Hospital in Novi Sad, Serbia assessed the quality of transmitted saved images by grading them from 1 to 5, where 1 was lowest and 5 was the highest grade. Reviewers graded transmitted images that were offered in uniform form. Grading was based on the presence or absence of anatomic landmarks (points and lines) used for morphologic classification of sonographic images according to Graf … Another reviewer graded transmitted real time video stream of DDH examination. Grading was based on diagnostic usefulness to confirm or exclude the DDH: can establish the diagnosis, need to repeat the examination, can't establish the diagnosis.”
	(3) Bias due to confounding	(3) Moderate	(3) All participants had normal US results. The results in the study may be biased by the exclusion of participants with abnormal US results: “Ultrasound examination of both hips revealed normal findings in all 25 examined babies.”
Nascimento et al. (33)	(1) Bias in measurement of outcomes	(1) Serious	(1) “…Because follow-up confirmatory echocardiograms were not considered, prevalence estimates may be biased upward, especially for handheld devices.”
	(2) Bias due to confounding	(2) Moderate	(2) Prevalence findings may be impacted by the differing demographic factors and other confounders between the different school groups: “…despite the multiple engagement strategies applied by the PROVAR study (markedly, the multiple educational strategies), student participation in public schools remained marginal, which may bias prevalence estimates … all consented children were consecutively included, without stratified sampling procedures, increasing the risk of bias associated with differences between groups (e.g., higher median age in private schools).”
Ross et al. (34)	(1) Bias due to confounding	(1) Moderate	(1) Historical control data was obtained from the period prior to the introduction of the US (June 2010). The validity of the historical control was assessed and Kruskal Wallis was used to determine if the data was consistent over time prior to June 2010 and after June 2012. Because there were no inconsistencies pre ultrasound and post ultrasound, confounding was believed not to have occurred and contributed to the # of deliveries or antenatal care visits. Additionally, # of delivers were obtained from a nearby government facility for the 2 years prior to and the 2 years following June 2010. The number of deliveries at this facility did not change prior to or after June 2010, further supporting the assumption that no confounding event occurred that affected the # of deliveries at the time ultrasound was introduced (June 2010)
Sekar and Vilvanathan (35)	(1) Bias in selection of the participants into the study	(1) No information	(1) No information was provided on how the clinicians determined “suspected congenital heart disease” in the patients they enrolled
	(2) Bias in measurement of outcome	(2) No information	(2) No information is provided about the pediatric cardiologist and whether a single cardiologist interpreted the images or if multiple cardiologists confirmed the findings
Sutherland et al. (36)	(1) Bias due to missing data	(1) Critical	(1) The authors initially included 6 ultrasound interpreters for the intervention group. However, 3 were *post-hoc* removed because of a failure to return reports or a significant delay in returning the reports, two measures that are part of the primary outcomes compared between the intervention and the control group.
Vinals et al. (37)	(1) Bias in selection of the participants into the study	(1) No information	(1) Two clinicians were chosen to participate in the study and were invited by email without clear indication how they were chosen
	(2) Bias in measurement of outcome	(2) Moderate	(2) Standardized image checklist was used but is not easily available in this publication or the reference publication and quality assessment is determined by a non-validated tool
Vinayak et al. (38)	(1) Bias in selection of the participants into the study	(1) No information	(1) Midwife participants volunteered to participate in the study which suggests a degree of interest and motivation and threatens external validity
	(2) Bias in measurement of outcome	(2) Moderate	(2) Standardized assessment tool was used by expert radiologists but not validated

### Summary Measures and Synthesis of Results

No summary measures were utilized in this narrative systematic review. The heterogeneity and quality of the studies prevented data from being combined or any formal measures of data consistency to be performed. Furthermore, due to the data quality, no meta-analysis was performed and there is no plan for a follow up meta-analysis.

## Results

### Search Results

**Figure 1:**
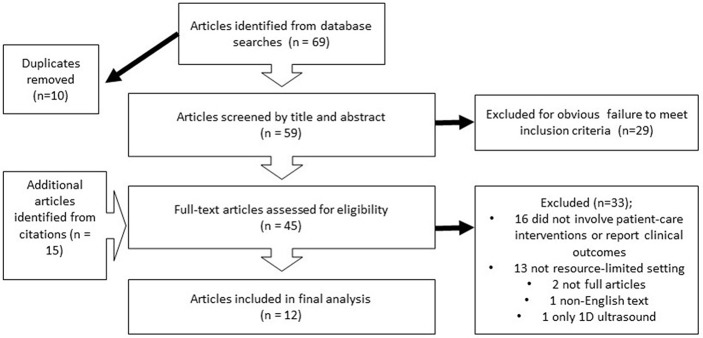
Search results: PRISMA diagram.

A literature search conducted in PubMed, MEDLINE, and Embase resulted in 69 articles with filters for English language and human subjects applied. Ten duplicates were removed for a total of 59 unique articles. After title and abstract review, 29 articles were removed due to failure to meet inclusion criteria. Fifteen additional articles were identified from citations. Of the remaining 45 articles that underwent full text evaluation, 16 were removed for either not involving a patient-care intervention or not reporting clinical outcomes, 13 were eliminated for failing to meet criteria for a resource-limited setting, and two abstracts without accompanying manuscripts were removed (one had an English title and abstract but foreign language full text; one utilized only 1D ultrasound). A total of 12 studies were included in the final analysis (27–38). A schematic of the study search and selection process is shown in Figure 1.

### Risk of Bias

We utilized the ROBINS-I tool provided by the Cochrane Collaboration to assess for bias within each individual study. The majority of the studies evaluated were non-randomized control trials, making the ROBINS-I tool most appropriate. The results of the bias evaluation are shown in Table 1. Explanation of the risk judgement categories are shown in Table 2. The heterogeneity of the studies and the lack of principal summary measures in the majority of the studies made any evaluation of between study bias using a tool such as GRADE meaningless (40).

**Table 2:** ROBINS-I risk categories.

**Risk of bias judgement**	**Explanation**
Low	The study is comparable to a well-performed randomized trial with regard to this domain
Moderate	The study is sound for a non-randomized trial with regard to this domain but cannot be considered comparable to a well-performed randomized trial
Serious	The study has some important problems with regards to this domain
Critical	The bias in this domain is too problematic to provide any useful evidence on the effects of the intervention from this study
No information	No information on which to base a judgement about risk of bias for this domain

### Synthesized Findings

The results of the included studies are summarized in Tables 3–5. The studies were conducted over diverse geographic locations. Of the twelve studies, six were in Africa (two in Togo, one in Mali, two in Uganda, one in Kenya), two in South Asia (India), two in South America (Chile and Brazil), one in Europe (Serbia), and one in the Caribbean (Dominican Republic). The study size ranged from 22 subjects to 12,048. Four of the studies were pilot or feasibility studies and two were single-site randomized trials with patients enrolled into either an experimental arm (telemedicine) or control (standard care that did not include telemedicine) arm.

**Table 3:** Study data for obstetrics-related papers.

**Authors (year)**	**Intervention location**	**Sample size**	**Real-time vs. asynchronous**	**US performer**	**Training provided**	**US interpreter**	**Tele-mentored**	**US type**	**Telemed platform**	**Primary results**
Bagayoko et al. (29)	Mali	215	Not specified	Midwifes and general physicians	3-week training	Not specified	No	Not specified	Laptops and low-bandwidth internet connections	(1) US helpful in diagnosis, frequently resulted in changed diagnosis and management; (2) Patients saved on average $25USD at telehealth site
Vinals et al. (37)	Chile	50	Asynchronous	Obstetricians	Not specified	Fetal echocardiography expert in Chile	No	STIC (Voluson 730 Expert series US scanner)	Broadband connection. Data received/stored in an external hard disk via USB connection	(1) Operators were successfully able to acquire images; (2) STIC datasets can be transmitted by the internet; (3) Fetal echo can be performed via a telemedicine link
Vinayak et al. (38)	Kenya	271	Asynchronous	Midwives	4-week training	2 radiologists with OB experience	No	Philips VISIQ tablet portable US	Mobile phone network and modem weblink	(1) Accuracy of images and measurements was 99.63%; (2) No additional reimaging required; (3) No reported image quality concerns from experts; (4) All patients felt safe, increased confidence, and better antenatal experience
Ross et al. (34)	Uganda	Unclear	Asynchronous	Midwives	3-day training	Referral hospital in Uganda	No	Not specified	Images compressed locally then transmitted via cellphone modem to remote server	(1) Increased number of attended deliveries after US implemented; (2) Increased number of antenatal care visits

**Table 4:** Study data for cardiology-related papers.

**Authors (year)**	**Intervention location**	**Sample size**	**Real-time vs. asynchronous**	**US performer**	**Training provided**	**US interpreter**	**Tele-mentored**	**US type**	**Telemed platform**	**Primary results**
Bhavnani et al. (30)	Bangalore, India	139	Asynchronous	Physicians	Yes, length not specified	Global consortium of 75 cardiologists	No	GE VScan	Cloud based system with broadband internet	Decreased time to referral for valvular interventions; lower probability of hospitalization or death
Sekar and Vilvanathan (35)	Aragonda, India	102	Real-time	Echo tech	Unknown	Pediatric cardiologist	Yes	Not specified	Very Small Aperture Terminal Satellite bandwidth; videoconferencing; satellite dish, high resolution camera, s-video cable, computer with webcam, monitor screen	Images were high quality; pathology ruled out in 49% of children; 51% were diagnosed with cardiac detect, and 29% referred for cardiac surgery
Nascimento et al. (33)	Minas Gerais, Brazil	12,048	Asynchronous	Nurse coordinators, biochemical & imaging technicians	12 weeks	Cardiologists	No	GE VIVID Q VScan	Dropbox© Cloud storage and downloaded for interpretation by dedicated VSCAN Gateway software	RHD overall prevalence was 4.0%; 29,695 children received educational curriculum

**Table 5:** Study data for non-obstetrics and non-cardiology-related papers.

**Authors (year)**	**Intervention location**	**sample size**	**Real-time vs. asynchronous**	**US performer**	**Training Provided**	**US interpreter**	**Tele-mentored**	**US type**	**Telemed platform**	**Primary results**
Adambounou et al. (28)	Togo	50	Both	Either physician or lay person using virtual navigation program	Unclear	Radiologists in France	Partial	GE Logiq 200	IP camera and remote access software	Adequate quality image transfer; satisfactory tele-diagnosis; low bandwidth requirement
Adambounou et al. (27)	Togo	22	Real-time	Physicians and technicians	Unclear	Radiologists in France	Yes	GE Logiq 200	video camera, internet, custom software for 3D reconstruction	Satisfactory diagnostic utility; good image quality; tele-mentored US possible
Epstein et al. (31)	Uganda	23	Real-time	Internal medicine resident	5 days	Radiologists and cardiologists in Israel	Yes	GE VScan	Commercially available video-chat software on cellular phones	Positive findings in 70% of cases; tele-US changed management 87% of cases
Sutherland et al. (36)	Veron, Dominican Republic	105	Asynchronous	Physician	2 months	6 volunteer radiologists in USA	No	Sonosite Titan	jpeg images sent by email and reports returned by email	Greater follow-up appointment attendance and shorter time to report in telemedicine group
Martinov et al. (32)	Zrenjanin, Serbia	25	Real-time	Sonographer	No additional training	Two sets of expert radiologists in USA and Serbia	No	Sonosite 180	Low bandwidth internet, commercially available software, video camera	Tele-diagnosis established from 62% of still images, 92% of videos; repeat scan needed in 8% of videos

Hospitalized patients were enrolled in five studies, outpatients in clinics were enrolled in six studies, and patients in both schools and clinics were enrolled in one study. The medical scope of the studies varied widely. Four studies primarily involved obstetrics (Table 3), three studies focused on cardiology (Table 4), and five studies focused on general practice or other specialties (Table 5). Five studies were designed as screening programs accomplished by tele-ultrasound.

Pocket and other portable ultrasound machines were the most commonly used ultrasound devices. In five studies, video images were captured via the ultrasound machine. Still images were captured in two studies, but the type of image capture was not specified in four studies. All studies required internet access. Four studies were designed to operate using low-bandwidth connections. Four studies used cameras to record images from the screen of the ultrasound machine, while the remaining eight studies sent ultrasound images without the use of camera recordings. Two studies relied on smartphones for image recording and transmission, and two studies utilized satellite for internet connectivity.

Substantial study diversity existed regarding who functioned as the bedside ultrasonographer and the level of expertise of the image interpreter. Physicians and midwives scanned patients in nine of the twelve studies. Scans were also obtained by trained sonographers, technicians, and non-healthcare professionals. Remote interpreters were dispersed around the globe. When the ultrasonographer and interpreter were in the same country, the interpreter was generally found at a large academic or major referral center. All image interpreters were trained radiologists or cardiologists. Tele-ultrasound was performed synchronously in four studies and asynchronously in six studies. Both methods were used in one study, and it was not specified which method was used in a final study. Remote tele-mentored ultrasonography was used in four of the twelve studies.

The most commonly reported outcome in the twelve studies was diagnostic utility of tele-ultrasound (eight studies), followed by assessment of image quality (six studies). Other reported outcomes include management impact of tele-ultrasound (four studies), telemedicine system requirements (four studies), impact on follow-up (four studies), data transmission reliability and quality (three studies), effectiveness of education program for ultrasound examiner (two studies), patient cost savings (two studies), time to referral or follow-up (two studies), likelihood of hospitalization or adverse outcome (one study), and patient satisfaction with tele-ultrasound (one study). All the studies showed generally positive results for the primary outcome measures that were assessed.

Cost was a commonly addressed concern throughout the included studies, with an emphasis on the need for affordable tele-ultrasound platforms to make its use feasible in low-resource settings. Several of the studies built telemedicine platforms using open-source or low-cost, commercially available software, over-the-counter hardware, and low-cost portable ultrasound machines in an effort to minimize costs. Two studies explored cost from the patients' perspective and found that the introduction of tele-ultrasound was associated with lower out-of-pocket costs for the patient, generally due to minimizing need for travel to larger medical centers with formal imaging capacity.

Our formal analysis of bias using the ROBINS-I tool identified significant potential risks of bias in most of the studies. The majority of the studies had both a risk of bias in measurement of outcomes (ten studies) and in the selection of participants into the study (nine studies). Due to inconsistencies in study outcomes reported, the lack of principal summary measures, and the low quality of the studies included which primarily encompassed technical feasibility and observational studies with few randomized control trials, no meta-analysis was performed.

## Discussion

### Summary of Main Findings

This systematic review suggests that tele-ultrasound performed in resource-limited settings can reliably produce satisfactory images with diagnostic utility that guide clinical management. According to the World Health Organization (WHO), imaging is needed for diagnosis in 20–30% of clinical cases and ultrasound and/or plain radiographs are sufficient for 80–90% of those cases. Yet, two-thirds of the world's population remains without access to medical imaging (14). Ultrasound, integrated into a telemedicine platform expands access to a safe, accessible, and affordable diagnostic imaging modality to populations in resource-limited settings.

Globally, ultrasound is a burgeoning diagnostic tool that often offers more insight into patient pathophysiology than the stethoscope. Thoracic ultrasound, as compared to chest radiography, has a high sensitivity and specificity for diagnosing cardiogenic pulmonary edema, pneumonia, COPD, pneumothorax, and pulmonary embolism in both the intensive care unit and the emergency department (41, 42). In fact, lung ultrasound is superior to chest radiograph in diagnosing pneumonia in the emergency department (43). In resource-limited settings, lung ultrasound was more sensitive and specific than chest radiograph to diagnose pneumonia (44). Furthermore, point-of-care ultrasound can be incorporated into a telemedicine platform and performed with relatively little training by non-physicians located at the bedside under the real-time guidance from ultrasound experts (20, 21, 45, 46). Thus, the use of RTMUS obviates the need for a bedside ultrasound expert to acquire images or a local expert to interpret them. RTMUS is particularly relevant in resource-limited settings in LMICs, where a scarcity of physicians often exists with expertise in ultrasound or with training in ultrasound-heavy subspecialties such as cardiology or obstetrics. Task-shifting ultrasound performance away from formally-trained sonographers and physicians to non-experts, while maintaining high quality imaging, helps establish a sustainable and cost-effective telemedicine program (47). This task-shifting also dramatically expands patient access to otherwise inaccessible subspecialists.

The studies included in this systematic review reinforce the concept that adequate ultrasound acquisition techniques can be taught in a remote tele-mentored manner. In cardiac ultrasound (Table 4), the high success rates for visualization of anatomic structures by non-experts allows for changes in medical management in the absence of a bedside physician. These changes include earlier treatment and appropriate escalation of care to tertiary centers (37). By utilizing non-experts as ultrasonographers, a larger population of patients gains access to ultrasonography as a diagnostic tool and to cardiology expertise. In this review, non-experts included physicians unfamiliar with a designated ultrasound approach, nurse research coordinators, a biomedical technician, and an imaging technician. Additional studies that did not meet the requirements for this review included custodians and medical interpreters as the non-experts performing the ultrasound (20). Collectively, these studies inform the conclusion that the quality of the ultrasound images obtained by non-experts are sufficient for interpretation by experts remotely.

Our literature review indicates that tele-ultrasound was frequently used in the field of cardiology (Table 4). Tele-ultrasound has demonstrated success in producing high quality, diagnostically significant images which alter management, decrease time to treatment, and provide more cost-effective care, especially when coupled with supporting data such as electrocardiogram, chest radiography, laboratory results, and clinical history (29, 30, 33, 35, 37, 48). In Aragonda, India, the use of remote tele-mentored echocardiography allowed for the diagnosis of pediatric cardiovascular pathology, resulting in a 29% referral for cardiac surgery based on those findings (35). In Bangladore, India, tele-ultrasound was used to assess times to treatment and long-term outcomes among children with structural heart disease. Images were collected in asynchronously and interpreted by a global consortium of cardiologists. Tele-ultrasound reduced the time to referral for valvular interventions and reduced the likelihood of both hospitalization and death (30). Though uncommon in high-income countries and likely underreported in low-income ones, rheumatic heart disease (RHD) is a major source of morbidity and mortality in LMICs (49). In the PROVAR study from Brazil, non-expert ultrasonographers successfully screened schoolchildren for RHD and images were interpreted by geographically-removed experts (33). Collectively, cardiology-based tele-ultrasound studies demonstrate the transformative potential of utilizing this imaging modality in a resource-limited setting as a tool to better understand the epidemiological impact of a disease and to improve disease management and outcomes.

Obstetrics is an additional medical specialty in which ultrasound is heavily utilized around the globe (50). Unfortunately, supply of ultrasound machines, sonographers, and radiologists in LMICs is very low. For example, only two radiologists work in Liberia (51). In an attempt to overcome such challenges, ultrasound training programs have taught non-experts either to independently perform obstetric ultrasounds to screen for high-risk pregnancies (52–55) or to utilize tele-ultrasound (29, 34, 37, 38). Of the multiple studies addressing the role of tele-ultrasound in resource-limited countries, the four included in this review focus on the obstetrics tele-ultrasound evaluation (Table 3). Ultrasonographers included physicians and midwives without prior obstetrics ultrasound training, but none of the obstetrics studies utilized RTMUS. Collectively, these studies concluded that ultrasound acquired accurate fetal structural views, allowed for the modification of perinatal care, and helped facilitate transfer to specialty centers when needed. Tele-ultrasound performed by a novice ultrasonographer prevented the need for additional re-imaging and yielded results available to the patient within 15 min. Image acquisition can be taught from a distance via the internet and a telemedicine platform is reliably able to transmit high quality images (29, 34, 37, 38).

Most of the studies included in this review implemented a brief training program for novice bedside ultrasonographers, regardless of the use of remote tele-mentored, real-time instruction. The training courses offered ranged from 3 days to 3 months. No correlation existed between the ultrasonographer's length of training and ability to adequately perform bedside ultrasound. Based on research not included in this systematic review, synchronous RTMUS can be successfully performed with <60 min of training (20, 21, 45, 46).

To date, we are unaware of any studies directly comparing synchronous to asynchronous telemedicine or tele-ultrasound. However, we believe an implicit benefit exists with using synchronous tele-ultrasound. Real-time image acquisition is well-suited to be combined with remote tele-mentoring to establish a hub-and-spoke paradigm whereby a single trained ultrasonographer can mentor numerous geographically removed ultrasound-naïve bedside providers to maximize the global reach of tele-ultrasound. By capitalizing on the concept of task-shifting inherent to RTMUS, any person located at the patient's bedside can function as the bedside ultrasonographer. Furthermore, real-time image acquisition and interpretation reduces delays in patient care and the need to return for follow up images, which may occur in an asynchronous point-and-store model of tele-ultrasound. Synchronous image acquisition also allows for real-time image quality control. As technology improves, wireless network and mobile phone access become more globally reliable, and commercially-available real-time audiovisual software (e.g., Skype, FaceTime) develop HIPAA-compliant platforms, the use of synchronous, RTMUS systems will be universally within reach.

The potential impacts of tele-ultrasound in LMICs are substantial with regard to the scope and breadth of both the numerous clinical areas (e.g., respiratory failure, hemodynamic compromise, procedural guidance) and the stakeholders (e.g., patients, providers, health systems) affected. The results of this systematic review, however, should be interpreted within the pre-established boundaries defined by the question we sought to answer using existing relevant studies. Specifically, this review addresses the feasibility of tele-ultrasound in LMICs and its clinical benefit to patients. Though certainly relevant to public health, this review was not intended to analyze the potential economic or workflow impacts of this technology on the health care providers or the health care system within each country. As public policy lies at the intersection of economic analysis and patient benefit, this systematic review cannot independently support changes to public policy but instead serves to further highlight the important clinical impact on patients.

### Limitations

Several limitations and biases impacted this review. It is possible that some articles were not assessed for eligibility due to the constraints of English language-only texts or articles not indexed on PubMed, MEDLINE, or Embase. Our goal was to capture those studies that utilized tele-ultrasound in resource-limited settings that involved direct patient care investigations and reported those outcomes accordingly. Excluding remote areas of high-income countries from our definition of resource-limited settings changed the available group of studies. While many important investigations have examined tele-ultrasound in remote settings of high-income countries (15, 16, 56), we chose to examine LMICs specifically in this review due to the fundamental differences in financial resources, healthcare personnel training and availability, health systems, and infrastructure that separate high-income countries from LMICs. Similar reasoning explains the exclusion of studies using robotic arm and virtual reality technologies. Several studies on the topic of tele-ultrasound in resource-limited settings were not included because they did not report a patient care intervention or meaningful clinical outcomes (20, 57–60). These studies were excluded because the goal of this review was to highlight those studies most germane to clinical practice, and studies in non-clinical environments that do not collect results relating to patient care are less easily clinically applicable.

The breadth of tele-ultrasound utilization was reflected in the marked heterogeneity of study designs. These varied designs led to different goals, outcomes, and reported data; moreover, their differences resulted in incomplete data when comparing studies. Many of the reported outcomes are related to technical feasibility or image quality interpretation and this evaluation is entirely subjective without the use of any standardized or validated measurement. This was compounded by the reality that the articles themselves were generally low quality and deemed as having a moderate to severe risk for bias, ranging from the selection of patients to be included in the study to bias regarding the selection of outcomes evaluated (Table 1). The high risk of bias in the majority of the included studies does limit the internal validity of the included studies. The missingness of reported data among the studies including the study design, patient selection, and participant selection limited the comparison of outcomes between studies. Control groups and randomization were rare. Nearly one-third of the studies were either pilot or feasibility studies. Collectively, this prevented substantial quantitative analysis of these studies and would certainly preclude any quantitative synthesis of the results into a meta-analysis. While all the articles reflected the promise of tele-ultrasound in resource-limited settings, the need for higher quality evidence in the future is obvious.

## Conclusions

The global burden of disease in resource-limited countries often outpaces the access to diagnostic modalities needed to identify disease and the availability of trained clinicians to treat disease. This supply-demand mismatch makes ultrasound a precious tool in resource-limited countries. Ultrasound is a low-cost, reliable, diagnostic tool which can be performed by minimally-trained bedside providers. Over the last quarter century, numerous advances have precipitated the feasibility and success of remote tele-ultrasound in resource-limited settings. Technologically speaking, ultrasound machines have become smaller, more portable and durable, and the relative cost has decreased dramatically. Smartphones are becoming more commonplace and seamlessly operate numerous software options which are capable of functioning as affordable handheld telemedicine platforms. Lastly, global connectivity is increasing, particularly wireless cellular and internet access. These advances, in concert, have made tele-ultrasound feasible and invaluable in resource-limited settings.

## Author Contributions

MAM performed initial systematic review and first draft of manuscript. SS helped with initial review and with manuscript edits. AS contributed to initial manuscript review of manuscripts included in systematic review and initial drafting of manuscript. AL edited manuscript and contributed to bias analysis. MTM oversaw project, involved in systematic review, and involved in drafting and revising the manuscript. NB performed bias analysis, revised paper to include bias analysis, and performed manuscript edits for second and third submission of paper.

### Conflict of Interest Statement

The authors declare that the research was conducted in the absence of any commercial or financial relationships that could be construed as a potential conflict of interest.
