# Supplementary material for: Tele-Ultrasound in Resource-Limited Settings: A Systematic Review
Source: Front Public Health. 2019 Sep 4;7:244. doi: 10.3389/fpubh.2019.00244 (PMC6738135; doi:10.3389/fpubh.2019.00244)
Supplement: Supplementary file 1 [file Data_Sheet_1.DOCX]

Supplementary Material

# Methods; Search Strategy; details

## PubMed/MEDLINE

((((((((telemedicine[Text Word] OR "telemedicine"[MeSH Terms]) OR ((remote medication[Title/Abstract] OR remote medicine[Title/Abstract]) OR (remote medication[Title/Abstract] OR remote medicine[Title/Abstract]) OR "distance medicine"[Title/Abstract])) OR (teleradiology[Title/Abstract] OR tele-radiology[Title/Abstract] OR tele radiology[Title/Abstract])) OR ((telepsychiatric[Title/Abstract] OR telepsychiatrist[Title/Abstract] OR telepsychiatrists[Title/Abstract] OR telepsychiatrists'[Title/Abstract] OR telepsychiatry[Title/Abstract] OR telepsychiatry'[Title/Abstract] OR telepsychiatry's[Title/Abstract]) OR (tele psychiatric[Title/Abstract] OR tele psychiatry[Title/Abstract]) OR (tele psychiatric[Title/Abstract] OR tele psychiatry[Title/Abstract]) OR tele pediatric[Title/Abstract] OR (telepediatric[Title/Abstract] OR telepediatrics[Title/Abstract]) OR tele pediatric[Title/Abstract] OR tele-intensivist[Title/Abstract])) OR ((tele education[Title/Abstract] OR tele-education[All Fields]) AND Title/Abstract[All Fields] AND medicine[Title/Abstract])) OR (ehealth[Title/Abstract] OR e-health[Title/Abstract] OR mhealth[Title/Abstract] OR m-health[Title/Abstract] OR e-medicine[Title/Abstract] OR e health[Title/Abstract] OR e medicine[Title/Abstract])) OR (telehealth[Title/Abstract] OR tele-health[Title/Abstract] OR tele health[Title/Abstract])) AND (((((("Poverty"[Mesh] OR "Medically Underserved Area"[Mesh]) OR "Developing Countries"[Mesh]) OR (under served[Title/Abstract] OR under-served[Title/Abstract])) OR (resource poor[Title/Abstract] OR resource-poor[Title/Abstract] OR resource limited[Title/Abstract] OR resource-limited[Title/Abstract])) OR (third world[Title/Abstract] OR third-world[Title/Abstract] OR developing world[Title/Abstract] OR developing-world[Title/Abstract] OR developing country[Title/Abstract] OR developing-country[Title/Abstract])) OR (low resource[Title/Abstract] OR low-resource[Title/Abstract] OR resource constrained[Title/Abstract] OR resource-constrained[Title/Abstract] OR resource limited[Title/Abstract] OR resource-limited[Title/Abstract]))) AND ("Ultrasonography"[Mesh] OR (ultrasound[Title/Abstract] OR tele ultrasound[Title/Abstract] OR tele-ultrasound[Title/Abstract] OR teleultrasonography[Title/Abstract] OR tele-ultrasonography[Title/Abstract] OR teleultrasound[Title/Abstract]))

## Embase

('echography'/exp OR 'ultrasound'/exp OR 'sonograph*':ab,ti OR 'tele-ultraso*':ab,ti OR 'teleultraso*':ab,ti OR 'ultrasound':ab,ti) AND ('resource limited setting'/exp OR 'poverty'/exp OR 'developing country'/exp OR 'medically underserved':ab,ti OR 'resource limited':ab,ti OR 'resource-limited':ab,ti OR 'resource poor':ab,ti OR 'third world':ab,ti OR 'developing country':ab,ti OR 'developing world':ab,ti OR 'low resource':ab,ti OR 'resource-constrained':ab,ti OR 'low-resource':ab,ti) AND ('telemedicine':ab,ti OR 'telemedicine'/exp/mj OR 'teleconsultation':ab,ti OR 'remote medicine':ab,ti OR 'distance medicine':ab,ti OR 'telehealth'/exp OR telehealth OR 'ehealth':ab,ti OR 'e-health':ab,ti OR 'mhealth'/exp OR mhealth OR 'm health' OR 'mobile health'/exp OR 'mobile health' OR (mobile AND ('health'/exp OR health)))
